# A critical examination of research narratives ‘rumours’ and passive community resistance in medical research

**DOI:** 10.1136/bmjgh-2021-007563

**Published:** 2022-04-22

**Authors:** Deborah Nyirenda, Salla Sariola, Nicola Desmond

**Affiliations:** 1 Social Science, Malawi-Liverpool-Wellcome Trust Clinical Research Programme, Blantyre, Malawi; 2 Faculty of Social Sciences, Sociology, University of Helsinki, Helsinki, Finland; 3 International Public Health, Liverpool School of Tropical Medicine, Liverpool, UK

**Keywords:** public health, health education and promotion, qualitative study

## Abstract

Several studies in Africa have reported effects of ‘rumours, misconceptions or misinformation’ on medical research participation and uptake of health interventions. As such, community engagement has sometimes been used for instrumental purposes to enhance acceptability of research or interventions and prevent ‘rumours’. This paper seeks to highlight the value of ongoing engagement with communities to understand research narratives ‘rumours’ reproduced in medical research. We demonstrate that ‘rumours’ are a form of divergent communication or local interpretation of medical research that needs critical attention, and we question the ethics of dismissing such divergent communication.

This paper draws on experiences from ethnographical research, which aimed to understand community engagement in medical research projects conducted in Malawi. We observed that even though community meetings were held to improve participation, ‘rumours’ about research influenced decision making. ‘Rumours’ presented local critiques of medical research, legitimate concerns informed by historical experiences and local conceptualisation of health. Structural inequalities, negative outcomes or absence of visible benefits following research participation informed unmet expectations, discontent with research and consequently passive resistance. The sociocultural context where participating research communities often rely on social networks for information nurtured propagation of these divergent perspectives to inform lay discourse around medical research.

We conclude that ongoing engagement, critical self-reflection and attempts to decode deeper meaning of ‘rumours’ throughout research implementation is necessary, to show respect and address community concerns expressed through ‘rumours’, enhance informed participation and adoption of future health interventions.

**Summary box**

- ‘Rumours’ about medical research have raised concerns among researchers to be detrimental to study participation.
- Research ‘rumours’ convey previous or lived experiences with medical research/interventions as well as critical views.
- Attributing research rumours to illiteracy raises ethical questions around research communication and conducting research with people whose capacity to understand medical research is undermined.
- There is need for ongoing engagement with community members to understand narratives around medical research in their socio-economic, political and cultural context and any differences with researchers’ views as divergent modes of communication.

## Introduction

Rumours about medical research have raised concerns among researchers to be detrimental to study participation. The idea that negative ‘rumours’ about medical research merely represent low scientific literacy, a so-called ‘deficit model’, has been critiqued by health communication scholars as inefficiency on the part of researchers to understand public concerns and build trust.^1^ To address this, participatory public engagement models are increasingly promoted to improve dialogue between scientists and public, build trust and improve the quality and relevance of scientific projects.^2^ Engaging the public or communities in medical research conducted in low-income settings is also promoted in the literature and international ethical guidelines.^3^ Historical mistrust towards medical research is one of the factors that necessitates communication between researchers and participants aimed to improve understanding and trust in medical research conducted in low-income settings such as sub-Saharan Africa (SSA).

Existing literature on the history of medical research in SSA shows that the introduction of medical research was often associated with negative experiences which affected community trust and fuelled negative narratives of health research.^4 5^ This was partly because the conduct of medical research was exploitative during colonial years and there were no ethical standards to protect participants from harm.^4 5^ Work by a historian Graboyes showed that fines, threats, physical beatings were at times used to enforce participation in medical research. Despite historical and geographical differences across SSA countries, research narratives about medical research around blood stealing, trade in body parts, surreptitious birth control and deliberate spreading of diseases, at different times, have affected community response to medical research and interventions.^6–10^ For instance, effects of ‘rumours’ in health interventions have resulted in boycotting of polio vaccine in Nigeria,^11^ community riots following mass drug administration of schistosomiasis medication in primary schools in Tanzania^8^ as well as refusals and withdrawals from medical research.^9^

More globally, the recent COVID-19 pandemic has demonstrated how ‘rumours’ influence uptake of COVID-19 preventive measures and COVID-19 vaccines.^12^ Some scholars have argued that rumours around COVID-19 signal global mistrust over uncertainties and risks of interventions such as COVID-19 vaccines.^13^ The perceived superiority of science which often dismisses lay opinion further contributes to the divide between scientists and the public leading to public mistrust.^14^ Following on existing literature that has discussed research ‘rumours’ as signalling poor relationships and mistrust between trial communities and researchers; this paper discusses research narratives framed as ‘rumours’ in two research communities as divergent communication that expresses local critiques as well as legitimate concerns among research participants and the wider community. We define divergent communication as community-driven perspectives of medical research or interventions, as well as reverse communication from community to researchers. We argue that labelling and dismissing such divergent perspectives of medical research as ‘rumours’ because they contradict medical knowledge and expectations is pejorative and fails to understand community perspectives which ultimately increases mistrust in medical research.

To understand divergent communication or lay discourses about medical research as a different form of ‘knowledge’ that emerges due to encounters with medical research, we draw on the literature on biomedicine and power. Building on the work of Foucault, biomedicine has been critiqued as authoritative because it exercises biopower by employing mechanisms to manage and influence individual or collective choices pertaining to health.^15^ Thus, biomedicine requires prescription of ‘norms’ and imposes truth about health that may interfere with individual choices. Foucault also states that ‘knowledge is power’^16^; and power allows medical researchers to produce knowledge or present medical ideas as ‘rational’ while dismissing indigenous knowledge or lay perspectives as being ‘irrational’. Thus, scientific rules can be used to further medical knowledge as valid while discrediting lay perspectives as irrational or ‘rumours’ and thereby sustaining hierarchies of knowledge.^17^ On the other hand, as power is exerted on groups or individuals, it generates resistance.^18^ Passive resistance may manifest through passive forms such as non-cooperation, silence, avoidance or deception as opposed to violent confrontations.^19^ Such forms of resistance may be masked with symbolic conformity,^20^ to resist medical research.

We draw on experiences from an ethnographical research project which aimed to understand community engagement through exploring case studies of medical research projects conducted in urban and rural settings in Malawi. We discuss how divergent communication revealed passive resistance and we question the ethics of dismissing such divergent communication as ‘rumours’. Passive resistance in this case does not imply passivity. Given the socioeconomic context where medical research presented opportunities to access better medical care and indirect benefits,^21 22^ passive resistance is a non-confrontational expression of agency to refuse research that does not address community concerns. Our two case studies included an urban case study involving the collection of nasal swabs from primary school children in an observation study and a rural case study implementing community-based malaria control interventions. The discussion in this paper focuses more broadly on prevailing research narratives from previous health interventions as well as emerging narratives during research implementation. DN, a female Malawian social scientist, who was not part of the study teams conducted ethnographical research as a participant observer in participating research communities. In addition, 43 in-depth interviews (IDIs) and 17 focus group discussions (FGDs) were conducted with research participants, non-research participants, community leaders, research staff and research volunteers in both sites to understand their experiences with research. The study was conducted in urban Blantyre and rural Chikwawa districts from June 2015 to July 2016. Details about the methods, analysis and findings have been presented elsewhere.^23^ This practice paper focuses on common research narratives which were cited in FGDs and IDIs with research participants, parents of children from participating schools, non-research participants, community leaders, research volunteers and research staff.

## ‘Rumours’ as expressions of concerns with previous medical research or interventions

Respondents’ (FGD and IDI participants) previous experiences with medical research practices or health interventions and the disconnect they experienced between promised health benefits and lived reality resulted in divergent communication. In the urban case study, FGD participants expressed concerns that researchers only communicated positive aspects of health interventions during community meetings to encourage participation without disclosing side effects, and this affected their trust in medical or health research and interventions. The most frequently cited example of selected side effects in FGDs with parents concerned an intervention where children were given schistosomiasis medication at school. Letters were sent to parents to inform them that children will be given medication at school without disclosing side effects of taking the medication on an empty stomach. Unfortunately, some students fainted after taking the medication and this was attributed to hunger by school and health authorities, but some parents refuted this. For example, a parent who participated in one of the FGDs said:

I was very disappointed that my child had taken a meal in the morning, but he came back home from school feeling weak and vomiting so much…they [the school] were wrong by not telling us the side effects beforehand … Male FGD participant, urban setting- FGD006

In the absence of an explanation from school officials about possible side effects experienced by children and how to avoid these, some parents suspected that the medication was designed to harm their children rather than to prevent them from schistosomiasis. The FGDs revealed that most participants did not consider schistosomiasis as a priority health problem, and they viewed medication as treatment rather than a means to prevent illness. It was therefore upsetting to such participants that the schools had given medication with serious side effects to children who were not sick. FGD participants also questioned why schools were giving out medication instead of clinics. Stories of children who fainted spread widely and confirmed parental concerns while reinforcing pre-existing suspicions that medical interventions were intended to cause death to reduce the country’s population growth. As a result, some parents discouraged their children from participating in future health research or interventions for fear of uncommunicated negative outcomes.

Since the urban case study involved taking nasal swabs from children in some of the schools that participated in the schistosomiasis intervention, this historical experience led to suspicion and passive resistance. Some parents deliberatively resisted the research by not responding to the invitation letters while children also deliberatively resisted by not delivering the invitation letters to parents. FGDs revealed that some parents and children were afraid to participate in the case study research due to previous experiences with the schistosomiasis intervention. Even though informed consenting procedures were followed, and children were allowed to give assent in the case study research; some children did not deliver invitation letters to avoid pressure from parents because they felt parents could override children’s decisions. This implies passive resistance or silent refusal^24^ as described in similar settings, because some parents and children did not openly refuse but they deliberately avoided research. In addition, FGD participants indicated that they passively resisted research because it was already approved by authorities and they could not report their concerns to them and be heard, as indicated in the following quote:

When research comes and we experience challenges, we have nowhere to turn to and complain…because it’s like the research was already approved…and It’s the same with medication…we get concerned when we encounter problems, but we have nowhere to complain to…that is why we sometimes shun research Male FGD participant, urban setting- FGD006.

Other parents, however, consented to have their children participate in the research because it did not involve medication. Choices to passively resist through non-participation were influenced by previous experiences where practices of medical research/interventions were at odds with local understanding of health or normal health service delivery as well as negative outcomes following medical research/interventions. Labelling such concerns as ‘rumours’ is a missed opportunity to understand community’s socioeconomic realities, their views and experiences with previous medical research/interventions that may shape future adoption of health interventions. This example also underscores the value of ongoing engagement with participating research communities to address divergent perspectives and prevent negative legacies about medical research/interventions that may affect future participation in medical research and interventions.

## ‘Rumours’ as expressions of critical views

While community meetings were used by the researchers to communicate study related information, local critiques of some research procedures circulated within social networks and impacted on decision making and passive resistance. An example of local critiques of medical research procedures pertained to a long-standing issue around drawing blood for research purposes and blood loss. Concerns around drawing blood ‘kupopa magazi’ (pumping blood) were widespread in both urban and rural settings and blood concerns have also been widely reported elsewhere across SSA.^4 6 7 25^ Some of the research narratives framed as ‘rumours’ expressed local critiques, particularly around medical research projects that were perceived to be taking frequent blood samples among sick people. Most of the FGD and IDI participants demonstrated an understanding that the body needed sufficient blood to function properly, and they associated frequent blood ‘pumping’ with ‘blood depletion’ (anaemia), ill health and death. As such, they attributed death of a research participant to anaemia when researchers drew blood samples regardless of the amount of blood taken. A narrative about a research participant who died while being followed up by researchers at their home to draw blood samples circulated in the urban community widely. Talking about this issue, one FGD participant commented:

My friend told me her experience of a child who participated in research. If they go [to the health facility] for instance today, they will take blood and if they go again next month, they will also take blood, until the child died. That’s why she was saying research is bad, her child died because they [researchers] took so much blood every now and then. Female FGD participant, urban setting-FGD003

Negative outcomes after research participation undermined participants’ trust in medical research and led to suspicions and concerns that medical research had ‘evil’ intentions to draw blood and cause death. Researchers on the other hand lumped such community concerns as ‘rumours’, as shown in the following quote:

Most of the times when we go to the community, people are afraid of researchers…they have fears that it’s the same issue of drawing blood…rumours are all over that the research is satanic (evil). FGD with research staff, urban setting-FGD007

In addition, some research participants who participated in FGDs in the rural case study also felt it was inappropriate to draw blood from children who were undernourished during the famine in 2016 when the research was being conducted. For instance, a male IDI participant in one village refused to enrol his child in research that involved drawing small blood samples because he feared that the child’s blood could ‘finish’. According to him, he felt that the researchers were insensitive and didn't have to draw blood samples when people were starving. Similar views were also expressed in FGDs as to why researchers needed blood from children who were starving. Research participants’ refusals to give blood samples were therefore local critiques about the risks to the child’s health and to further impoverish local people.

At times, critical views concerning medical research reflected misunderstanding between research procedures, clinical assessment and access to treatment. FGD participants particularly in the urban case study indicated that they participated in research to access individual benefits such as clinical assessment, treatment, monetary compensation or reimbursement because they were exposed to previous medical research that offered these. Negative outcomes such as death or ill health following research participation in a setting where morbidity and mortality were high often led some respondents to associate medical research with ill intentions. FGD participants expressed disappointment with the extractive nature of medical research where they saw researchers drawing blood, and the patient did not get better. For example, a mother who participated in previous research involving blood samples commented:

… I asked myself, if they draw blood from my child frequently, will the child get better? They keep coming and coming and each time they come, they draw blood…so will the child get better? That is why I decided to withdraw [from the research]. Mother of a research participant, urban setting-IDI017

While some research staff in both case studies attributed these concerns around blood samples to illiteracy, the negative experiences particularly among people whose family members or friends experienced negative outcomes spread reluctance to participate among people in their wider social networks. Labelling such concerns as ‘rumours’ or superstition, is a form of ‘othering’ that exoticises local understanding and negates research participants’ perspectives as less worthy of consideration. Failure to consider research participants’ concerns can escalate how participants and wider community members perceive medical research, reinforce critical opinions and passive resistance. Moreover, attributing research participants’ concerns to ignorance raises questions about the extent to which research and its relevance has been communicated in ways that participants can understand. This also raises potential ethical questions around conducting research in low literacy settings with people whose capacity to comprehend medical research is deemed low.

## ‘Rumours’ as enablers of positive behaviour change

On the other hand, shared experiences of benefits of medical interventions enhanced informed decision making to adopt some of the interventions in the rural case study. The engagement of few residents as research volunteers also facilitated ongoing engagement between researchers and community to elicit and address emerging community concerns. Research participants’ experiences with research were usually circulated beyond the intervention villages in social gatherings; firsthand or secondhand testimonies from family or peers informed their decisions to participate in medical research or adopt health interventions. Since most of the participating research communities had a history of oral tradition and relied on verbal communication, research volunteers served as reliable sources of study information, and they were more trusted than outsiders.

In the rural case study, some research participants who participated in FGDs were initially scared of mosquito traps and passively resisted the malaria control interventions because of the links between research and evil. For example, one FGD participant commented:

When they brought it to my house…I told them, it looks like something used by traditional healers, do you want to take our blood? They explained that they just wanted to find out if there were mosquitoes in the house…but it looked like a calabash, and it had something like a bell at the centre…it wasn’t different from what traditional healers use. It also had a gallon, and there was like blood (molasses) inside… Male FGD participant, rural setting-FGD016

To assess whether the population of mosquitoes was reducing, field workers were leaving mosquito traps in selected households to stay overnight. Most of the respondents however believed that evil things or witchcraft (ufiti) took place mysteriously at night to cause ill health, disabilities, or death. In addition, the mosquito traps used molasses to attract mosquitoes, but some respondents thought the molasses was blood that was mysteriously drawn from household members. FGD participants expressed initial fears that the strange looking mosquito trap would suck their blood mysteriously while sleeping and they passively resisted the mosquito traps in their homes. They however claimed that initial fears that mosquito traps would suck human blood were cleared when none of the research participants reported negative experiences. Research staff and FGD participants claimed that information about research participant’s experiences of the malaria control interventions spread beyond intervention villages. For instance, some people in non-intervention villages started covering their windows with bed nets or wire mesh because they heard positive experiences that covering windows prevented mosquitoes and malaria. Thus, research participant’s first-hand experiences or anecdotal evidence within their social networks impacted on decision making either positively or negatively. This shows that decision making around research participation was informed by evolving encounters with medical research which were often validated through their social networks, hence emphasising the importance of ongoing engagement.

## Conclusion

This paper has discussed research narratives around medical research/interventions as a form of divergent communication. Divergent communication emerged due to historical experiences with medical research/interventions, critical views generated from local understanding of health, and common-sense reflections about certain medical research procedures. The social context where people often rely on social networks for information propagated wide circulation of divergent communication in participating research communities and passive resistance.

We argue that labelling research participants’ concerns as ‘rumours’ exoticises local knowledge, reflects a failure to engage with local knowledge and counteracts coproduction of knowledge promoted in community engagement literature. This also raises concerns on conducting research with people whose capacity to understand medical research is deemed low. We conclude that one off community engagement meetings do not suffice to address the legacy of negative research narratives or elicit emerging concerns that may ultimately be detrimental to future research participation and adoption of health interventions.

We propose that a better way of understanding the different epistemic forms circulating around medical research would be to understand research participant’s views in their socioeconomic, political and cultural context and any differences with researchers’ views as divergent modes of communication. Ongoing engagement with communities throughout study implementation and critical self-reflection is necessary to decode and respond to collective concerns expressed through ‘rumours’. Since people in the rural case study relied on verbal information from social networks, we found out that engagement of few residents as research volunteers improved access to information and encouraged discussion of study information beyond community meetings. This approach also fitted well with the closely knit cultural context in the rural setting and pre-existing social norms where people accessed information from social networks.

## Data Availability

Data are available on reasonable request.
